# What I wish I’d learned as an orthodontic trainee: an online survey of British Orthodontic Society members concerning postgraduate training experiences

**DOI:** 10.1177/1465312520904367

**Published:** 2020-02-13

**Authors:** Graham R Oliver, Christopher D Lynch, Padhraig S Fleming

**Affiliations:** 1 Bristol Dental School, University of Bristol, Bristol, UK; 2 University Dental School & Hospital/Cork University Dental School & Hospital, Cork, Ireland; 3 Institute of Dentistry, Barts and The London, UK

**Keywords:** education, postgraduate, orthodontics

## Abstract

**Objective:**

To survey the opinion of recently qualified and established orthodontists on the perceived value of their training and to identify specific areas which which were considered to be deficient, adequately covered or focussed on excessively.

**Design:**

Descriptive cross-sectional survey

**Setting:**

Online electronic questionnaire.

**Participants:**

Members of the British Orthodontic Society (BOS).

**Methods:**

An electronic questionnaire was circulated to members of the BOS focusing on dental education history, and opinions concerning orthodontic teaching generally and specific clinical and non-clinical subjects. Information was also obtained in terms of possible need for improvement, modification or removal of teaching on focused academic and clinical aspects.

**Results:**

A total of 217 responses were received from 1080 emailed invitations resulting in a response rate of 20.1%. Respondents were generally satisfied with their training both in relation to theoretical, academic and practical aspects. However, training was regarded as deficient by some respondents in respect of temporary anchorage devices (38%), bonded retainers (6%), experience with lingual appliances (47%), removable aligners (44%), inter-proximal reduction (24%) and adult orthodontics (16%), working with therapists (32%), and NHS contracts (47%) and commissioning (47%).

**Conclusion:**

The overall satisfaction of BOS members with postgraduate orthodontic training is generally high, although both recently qualified and established practitioners emphasised the need for better exposure to training in specific practical aspects and practice management within the NHS.

## Introduction

Training in orthodontics dates from the infancy of the specialty led by Edward H Angle who founded the Angle School of Orthodontia in St Louis, Missouri. In the UK, George Northcroft and colleagues formed the British Society for the Study of Orthodontia (BSSO) in 1907 which eventually became one of the five societies to unify to form the British Orthodontic Society (BOS) in 1994. The BSSO recommended that orthodontics be set up as a one-year postgraduate programme delivered in dental schools or specialist centres. Qualifications in orthodontics were introduced in the UK in 1948 when the Dental Committee of the Royal College of Physicians and Surgeons of Glasgow sanctioned the development of a diploma which was subsequently approved by the Dental Board of the United Kingdom. The first sitting of this exam was in 1949 and was followed soon after by a Diploma from the Royal College of Surgeons of England in 1954. In the late 1980s, Memberships in Orthodontics of the Royal Colleges began to supersede the original diploma. Two-year university Masters level degrees were first offered by the Welsh National School of Medicine in 1974 (Robertson, 1973) before being introduced by other dental schools across the UK.

Currently, orthodontic training involves a three-year university-based training programme requiring completion of a level 7 or 8 degree (Master’s degree or Professional Doctorate, respectively). Within the UK, each year approximately 30 students enter salaried NHS training posts with linked National Training Numbers (NTNs). The Specialty Advisory Committee (SAC) in orthodontics is responsible for overseeing the orthodontic postgraduate curriculum against which the Royal College Examinations are mapped, subsequently being approved by the General Dental Council (GDC). The latest curriculum was published in 2010 being geared at providing trainees with ‘the appropriate knowledge, attitudes and skills of a Specialist Orthodontist’ (The Joint Committee for Postgraduate Training in Dentistry and The Specialty Advisory Committee in Orthodontics, 2010). Orthodontic training programmes are designed to reflect patterns of care with most training programmes focusing on treatment with fixed appliances (O’Brien and Spencer, 2015). Orthodontic systems continue to develop with a range of variants including removable aligner and lingual systems now taking an increasing market share; however, these treatments are not typically offered within the NHS.

A previous survey of trainees completing UK-based programmes highlighted that 20% felt training did not meet expectation (Keith et al., 1997). More recently, a survey of UK-based and international postgraduates reported a 76% satisfaction rate (Oh and Chadwick, 2016). Respondents were generally satisfied with their caseload (78.4%); however, concerns were raised among UK trainees about the value for money as well inability to influence the delivery of teaching. Outside of the UK, a survey of trainees in Turkey reported a lower satisfaction rate (58%) with programmes deemed deficient in providing care to underserviced populations and disabled patients as well as in terms of exposure to multidisciplinary treatments (Usumez et al., 2013). Higher levels of satisfaction of 86% and 76% have been reported in similar surveys in Canada and the USA, respectively (Noble et al., 2009a, 2009b).

Notwithstanding this, there is currently no information regarding the perceived value of orthodontic training among qualified orthodontists in the UK, nor is there any information in relation to specific areas of the curriculum. As such, it is of interest to stakeholders in postgraduate orthodontic education to better understand the opinions of all stakeholders. Our aims were therefore to survey the opinion of recently qualified and established orthodontists on the perceived value of their training and to identify specific areas which they believed to be deficient, adequately covered or over-emphasised.

## Methods

This was a descriptive, cross-sectional study in which an online questionnaire was distributed to members of the BOS. The 12-item questionnaire was developed based on a previous survey into opinions concerning undergraduate dental education (Oliver et al., 2016). Ethical approval was provided by Queen Mary University of London, Ethics of Research Committee (QMREC2046) with prior approval from the BOS, Clinical Governance Committee. Members of the Consultant Orthodontic Group (COG), Community Group (CG), Orthodontic Specialists Group (OSG), University Teachers Group (UTG), Practitioner Group (PG) and Post-Certificate of Completion of Specialist Training (Post-CCST) trainees of the Trainee Grades Group (TGG) of the BOS were invited to participate in the survey via an initial email in March 2019. Two reminder emails were sent thereafter at three-weekly intervals. The survey was open for 10 weeks from March until May 2019. The survey was administered, and results collected using Online Surveys (JISC, Bristol, 2019).

Questions were asked to gain an understanding of the respondents’ dental education history and current place of work. Opinions were sought on specific areas of clinical and non-clinical training, areas of orthodontics or training where exposure or experience could be increased or reduced, as well as ascertaining how well postgraduate training prepared former students for working as a specialist orthodontist (Appendix 1).

Results were assessed for the group as a whole, with further comparison between recent graduates (< 10 years since graduation) and ‘established practitioners’ (qualified ⩾ 10 years). Statistical analysis included demographic data allied to Chi-squared tests to assess possible differences between recent and established practitioners. In cases of insufficient data, Fisher’s exact test was used with a *P* value < 0.05 representing statistical significance. Free-text responses were also coded and described.

## Results

A total of 217 responses were received from 1080 emails on the BOS mailing list, representing a 20.1% response rate. The majority of respondents (n = 140; 64.5%) were ‘established orthodontists’ having completed orthodontic training before 2009, while ‘recent orthodontic graduates’ comprised a smaller proportion (n = 71; 32.3%). Six respondents failed to provide this information.

Of the respondents, 55% were female (n = 119) and 43.5% were male (n = 95). One respondent was aged < 30 and 20 were aged > 60 years (Table 1). Respondents had primarily completed both their undergraduate (93.5%) and orthodontic (95%) training within a UK or Irish university (Table 2). Of respondents, 66% (n = 143) work within a specialist dental practice setting, while 37% (n = 80) were NHS hospital consultants. Fifteen (10%) respondents were undertaking a PhD or Post-CCST training with some respondents working in multiple settings.

**Table 1.** Demographic characteristics of respondents (n = 217).

	Recent graduates (n = 70)	Experienced orthodontists established practitioners (n = 141)	Total (n = 217)
*Gender*			
Male	26 (36.5)	66 (47)	95 (43.5)
Female	45 (63.5)	72 (51.5)	119 (55)
Undisclosed	0 (0)	2 (1.5)	3 (1.5)
*Age (years)*			
< 30	1 (1.5)	0 (0)	1 (0.5)
30–40	54 (76)	4 (3)	59 (27)
41–50	14 (19.5)	65 (46)	80 (37)
51–60	2 (3)	50 (36)	56 (26)
> 60	0 (0)	20 (14)	20 (9)
Undisclosed	0 (0)	1 (1)	1 (0.5)

Values are given as n (%).

**Table 2.** Information pertaining to the location, decade and level of qualification in orthodontics.

Orthodontic training	Recent graduates (n = 70)	Experienced orthodontists established practitioners (n = 141)	Total (n = 217)
UK & Ireland	70 (100)	134 (95)	207 (95)
Other EEA	0 (0)	1 (0.75)	1 (0.5)
Other	0 (0)	2 (1.25)	2 (1)
Undisclosed	0 (0)	4 (3)	7 (3)
2010–2019	65 (91.5)	0 (0)	65 (30)
2000–2009	6 (8.5)	71 (51)	77 (35.5)
1990–1999	0 (0)	48 (34)	48 (22)
1980–1989	0 (0)	21 (15)	21 (9.5)
Undisclosed			6 (3)
Diploma	1 (1.5)	9 (6.5)	12 (6)
Masters (Taught)	34 (48)	80 (57)	116 (54)
Masters (Research)	21 (29.5)	38 (27)	60 (28)
Doctorate (Taught)	8 (11)	1 (0.5)	10 (5)
Doctorate (Research)	6 (8.5)	8 (6)	14 (6.5)
Undisclosed	1 (1.5)	4 (3)	5 (2)

Values are given as n (%).

EEA, European Economic Area.

## Knowledge, theory and diagnosis

There was general satisfaction with the depth of training in the areas of research and critical appraisal (68%, n = 148), clinical governance (60%, n = 130), oral and dental health education (83%, n = 180), and epidemiology (67%, n = 146). Psychology, however, had the lowest satisfaction rate (n = 70, 32%) with 65% (n = 141) wishing they had learned more or feeling their training was deficient (Table 3).

**Table 3.** Levels of satisfaction with teaching of a range of theoretical, diagnostic and treatment aspects among the sample (n = 217).

	**Recent graduates**					**Established practitioners**					**Total**					*P* **value (Chi-squared test)**
	I learned more than I needed to	I learned the right amount	I wish I learned more	My training was deficient	No response	I learned more than I needed to	I learned the right amount	I wish I learned more	My training was deficient	No response	I learned more than I needed to	I learned the right amount	I wish I learned more	My training was deficient	No response	
Research and critical appraisal	11 (15)	45 (63)	8 (11)	7 (10)	0 (0)	9 (6)	99 (71)	28 (20)	2 (1)	2 (1)	20 (9)	148 (68)	38 (18)	9 (4)	2 (1)	0.000*
Clinical governance	2 (3)	51 (72)	14 (20)	4 (6)	0 (0)	2 (1)	77 (55)	38 (27)	20 (14)	2 (3)	4 (2)	130 (60)	55 (25)	24 (11)	4 (2)	0.000*
Oral and dental health education	1 (1)	55 (77)	12 (17)	2 (3)	1 (1)	4 (3)	120 (86)	10 (7)	4 (3)	2 (1)	6 (3)	180 (83)	22 (10)	6 (3)	3 (1)	0.046
Psychology	1 (1)	28 (39)	31 (44)	11 (15)	0 (0)	1 (1)	37 (26)	75 (54)	23 (16)	4 (3)	2 (1)	70 (32)	106 (49)	35 (16)	4 (2)	0.074
Epidemiology	5 (7)	43 (61)	13 (18)	9 (13)	1 (1)	4 (3)	98 (70)	26 (19)	10 (7)	2 (1)	9 (4)	146 (67)	39 (18)	19 (9)	4 (2)	0.095
Aetiology of malocclusion	1 (1)	68 (96)	2 (3)	0 (0)	0 (0)	4 (3)	131 (94)	4 (3)	0 (0)	1 (1)	5 (2)	204 (94)	7 (3)	0 (0)	1 (0)	0.701^†^
Clinical diagnosis skills	1 (1)	64 (90)	6 (8)	0 (0)	0 (0)	6 (4)	124 (89)	7 (5)	2 (1)	1 (1)	7 (3)	193 (89)	14 (6)	2 (1)	1 (0)	0.312^†^
Facial and dental aesthetics	2 (3)	40 (56)	24 (34)	5 (7)	0 (0)	4 (3)	88 (63)	40 (29)	6 (4)	2 (1)	6 (3)	134 (62)	64 (29)	11 (5)	2 (1)	0.450
Radiology	1 (1)	59 (83)	8 (11)	3 (4)	0 (0)	2 (1)	122 (87)	15 (11)	0 (0)	1 (1)	3 (1)	187 (86)	23 (11)	3 (1)	1 (0)	0.217
Cephalometry	7 (10)	58 (82)	5 (7)	1 (1)	0 (0)	19 (14)	115 (82)	5 (4)	0 (0)	1 (1)	26 (12)	179 (82)	10 (5)	1 (0)	1 (0)	0.211
3D imaging	3 (4)	17 (24)	39 (55)	12 (17)	0 (0)	0 (0)	22 (16)	71 (51)	35 (25)	12 (9)	3 (1)	40 (18)	114 (53)	47 (22)	13 (6)	0.010
Treatment planning	1 (1)	60 (85)	7 (10)	3 (4)	0 (0)	7 (5)	116 (83)	14 (10)	2 (1)	1 (1)	8 (4)	180 (83)	23 (11)	5 (2)	1 (0)	0.011
*Biology*																
Cell and molecular biology	13 (8)	51 (72)	5 (7)	2 (3)	0 (0)	30 (21)	95 (68)	10 (7)	3 (2)	2 (1)	43 (20)	152 (70)	15 (7)	5 (2)	2 (1)	0.822
Embryology	12 (17)	49 (69)	7 (10)	3 (4)	0 (0)	22 (16)	104 (74)	11 (8)	2 (1)	1 (1)	34 (16)	159 (73)	18 (8)	5 (2)	1 (0)	0.433
Dental growth and development	4 (6)	58 (82)	5 (7)	4 (6)	0 (0)	7 (5)	122 (87)	8 (6)	2 (1)	1 (1)	11 (5)	186 (86)	13 (6)	6 (3)	1 (0)	0.281
Craniofacial growth and development	5 (7)	54 (76)	8 (11)	4 (6)	0 (0)	10 (7)	117 (84)	10 (7)	2 (1)	1 (1)	15 (7)	177 (82)	18 (8)	6 (3)	1 (0)	0.145
*Treatments and appliances*																
Interceptive treatment	0 (0)	47 (66)	21 (30)	3 (4)	0 (0)	1 (1)	112 (80)	25 (18)	1 (1)	1 (1)	1 (0)	165 (76)	46 (21)	4 (2)	1 (0)	0.043^†^
Removable appliances	1 (1)	59 (83)	10 (14)	1 (1)	0 (0)	12 (9)	118 (84)	8 (6)	1 (1)	1 (1)	15 (7)	181 (83)	18 (8)	2 (1)	1 (0)	0.000*
Functional appliances	0 (0)	67 (94)	4 (6)	0 (0)	0 (0)	11 (8)	120 (86)	8 (6)	0 (0)	1 (1)	11 (5)	192 (88)	13 (6)	0 (0)	1 (0)	0.013^†^
Extra-oral appliances	6 (8)	47 (66)	14 (20)	4 (6)	0 (0)	13 (9)	114 (81)	9 (6)	3 (2)	1 (1)	20 (9)	165 (76)	24 (11)	7 (3)	1 (0)	0.002
Pre-adjusted edgewise appliances	2 (3)	66 (93)	3 (4)	0 (0)	0 (0)	6 (4)	124 (89)	6 (4)	2 (1)	2 (1)	8 (4)	194 (89)	11 (5)	2 (1)	2 (1)	0.882^†^
Tip-edge appliances	9 (13)	28 (39)	22 (31)	12 (17)	0 (0)	24 (17)	73 (52)	23 (16)	12 (9)	8 (6)	33 (15)	106 (49)	46 (21)	24 (11)	8 (4)	0.001
Begg appliances	6 (8)	31 (44)	16 (23)	16 (23)	2 (3)	25 (18)	82 (59)	15 (11)	12 (9)	6 (4)	33 (15)	116 (53)	32 (15)	28 (13)	8 (4)	0.000*
Aligner appliances	0 (0)	5 (7)	27 (38)	39 (55)	0 (0)	1 (1)	19 (14)	50 (36)	55 (39)	15 (11)	1 (0)	26 (12)	79 (36)	96 (44)	15 (7)	0.102^†^
Lingual appliances	0 (0)	5 (7)	25 (35)	41 (58)	0 (0)	1 (1)	15 (11)	48 (34)	58 (41)	18 (13)	1 (0)	22 (10)	75 (35)	101 (47)	18 (8)	0.239^†^
Removable retention appliances	1 (1)	66 (93)	2 (3)	2 (3)	0 (0)	5 (4)	126 (90)	5 (4)	2 (1)	2 (1)	7 (3)	196 (90)	8 (4)	4 (2)	2 (1)	0.231
Fixed/Bonded retention appliances	0 (0)	54 (76)	11 (15)	5 (7)	1 (1)	4 (3)	102 (73)	24 (17)	8 (6)	2 (1)	5 (2)	161 (74)	35 (16)	13 (6)	3 (1)	0.424^†^
Temporary anchorage devices	0 (0)	21 (30)	25 (35)	25 (35)	0 (0)	1 (1)	20 (14)	48 (34)	55 (39)	16 (11)	1 (0)	42 (19)	75 (35)	82 (38)	17 (8)	0.069^†^
Inter-dental enamel reduction	0 (0)	25 (35)	30 (42)	16 (23)	0 (0)	1 (1)	52 (37)	45 (32)	35 (25)	7 (5)	1 (0)	80 (37)	76 (35)	53 (24)	7 (3)	0.512^†^
*Multidisciplinary care*																
Orthodontics and periodontal disease	0 (0)	33 (46)	33 (46)	5 (7)	0 (0)	1 (1)	88 (63)	44 (31)	7 (5)	0 (0)	1 (0)	125 (58)	79 (36)	12 (6)	0 (0)	0.053^†^
Management of impacted/ectopic teeth	2 (3)	63 (89)	4 (6)	2 (3)	0 (0)	4 (3)	122 (87)	12 (9)	2 (1)	0 (0)	6 (3)	190 (88)	17 (8)	4 (2)	0 (0)	0.523
Management of hypodontia	2 (3)	57 (80)	10 (14)	2 (3)	0 (0)	3 (2)	94 (67)	39 (28)	4 (3)	0 (0)	5 (2)	156 (72)	50 (23)	6 (3)	0 (0)	0.001
Management of obstructive sleep apnoea	1 (1)	23 (32)	29 (41)	18 (25)	0 (0)	4 (3)	33 (24)	55 (39)	35 (25)	13 (9)	5 (2)	58 (27)	86 (40)	54 (25)	14 (6)	0.266
Management of facial deformity/Orthognathic	4 (6)	49 (69)	16 (23)	2 (3)	0 (0)	7 (5)	106 (76)	26 (19)	1 (1)	0 (0)	11 (5)	160 (74)	43 (20)	3 (1)	0 (0)	0.393
Adult orthodontics	0 (0)	29 (41)	28 (39)	14 (20)	0 (0)	1 (1)	57 (41)	62 (44)	19 (14)	1 (1)	1 (0)	89 (41)	92 (42)	34 (16)	1 (0)	0.533^†^
Trauma in orthodontics	0 (0)	34 (48)	29 (41)	8 (11)	0 (0)	0 (0)	77 (55)	50 (36)	13 (9)	0 (0)	0 (0)	115 (53)	79 (36)	23 (11)	0 (0)	0.642^†^
*Risks and benefits*																
Psychological/Quality of life benefits	1 (1)	47 (66)	19 (27)	4 (6)	0 (0)	2 (1)	78 (56)	48 (34)	7 (5)	5 (4)	3 (1)	130 (60)	68 (31)	11 (5)	5 (2)	0.278
Dental health benefits	0 (0)	61 (86)	9 (13)	1 (1)	0 (0)	2 (1)	126 (90)	8 (6)	2 (1)	2 (1)	3 (1)	191 (88)	18 (8)	3 (1)	2 (1)	0.240^†^
Orthodontically induced inflammatory root resorption	1 (1)	54 (76)	15 (21)	1 (1)	0 (0)	1 (1)	106 (76)	29 (21)	3 (2)	1 (1)	3 (1)	164 (76)	45 (21)	4 (2)	1 (0)	0.865^†^
Decalcification/demineralisation	3 (4)	60 (85)	8 (11)	0 (0)	0 (0)	4 (3)	131 (94)	4 (3)	0 (0)	1 (1)	8 (4)	195 (90)	13 (6)	0 (0)	1 (0)	0.063^†^
Relapse	2 (3)	59 (83)	8 (11)	2 (3)	0 (0)	2 (1)	112 (80)	22 (16)	2 (1)	2 (1)	5 (2)	175 (81)	30 (14)	5 (2)	2 (1)	0.356^†^
Temporomandibular joint dysfunction	1 (1)	48 (68)	19 (27)	3 (4)	0 (0)	5 (4)	86 (61)	39 (28)	8 (6)	2 (1)	6 (3)	137 (63)	61 (28)	11 (5)	2 (1)	0.216
*Orthodontics in the NHS*																
Working with orthodontic therapists	0 (0)	23 (32)	27 (38)	21 (30)	0 (0)	0 (0)	27 (19)	51 (36)	45 (32)	17 (12)	0 (0)	52 (24)	78 (36)	69 (32)	18 (8)	0.285^†^
Contracts	0 (0)	6 (8)	31 (44)	34 (48)	0 (0)	1 (1)	16 (11)	44 (31)	65 (46)	14 (10)	1 (0)	23 (11)	76 (35)	102 (47)	15 (7)	0.001
Commissioning	0 (0)	6 (8)	30 (42)	34 (48)	1 (1)	1 (1)	17 (12)	43 (31)	65 (46)	14 (10)	1 (0)	24 (11)	74 (34)	102 (47)	16 (7)	0.346^†^
Primary care orthodontics	1 (1)	19 (27)	29 (41)	22 (31)	0 (0)	0 (0)	67 (48)	38 (27)	30 (21)	5 (4)	1 (0)	89 (41)	69 (32)	53 (24)	5 (2)	0.000*
Secondary care orthodontics	1 (1)	49 (69)	12 (17)	9 (13)	0 (0)	3 (2)	91 (65)	26 (19)	13 (9)	7 (5)	4 (2)	144 (66)	39 (18)	23 (11)	7 (3)	0.640

Values are given as n (%).

* *P* < 0.001.

† In cases of insufficient data, Fisher’s exact tests were used.

Respondents were satisfied with theoretical knowledge and diagnostic procedures. A total of 204 (94%) respondents believed that they had received the right amount of training concerning aetiology of malocclusions. Clinical diagnosis skills were also well received with 89% (n = 193) satisfied with their training. Similar levels of satisfaction were reported in relation to radiology (86%, n = 187), cephalometry (82%, n = 179) and treatment planning skills (83%, n = 180). Lower satisfaction was associated with three-dimensional imaging techniques with 53% (n = 114) wishing they learned more and 22% (n = 47) feeling their training was deficient in this respect.

## Treatments and appliances

Satisfaction was highest with training in relation to removable (83%, n = 181), functional (88%, n = 192) and pre-adjusted edgewise appliances (89%, n = 194), and removable retainers (90%, n = 196). Only a small percentage wished they learned more in relation to removable appliances (8%, n = 18), functional appliances (6%, n = 13), pre-adjusted edgewise appliances (5%, n = 11) and removable retainers (4%, n = 8). Training in relation to fixed retainers received 74% (n = 161) satisfaction with 16% (n = 35) wishing they learned more (Table 3).

There were also mixed responses to training in inter-dental enamel reduction with 37% (n = 80) learning the right amount, 35% (n = 76) wished they learned more and 24% (n = 53) thought their training was deficient. Satisfaction with training in temporary anchorage devices was also relatively low with slightly greater satisfaction among recent graduates at 30% (n = 42) compared to established practitioners at 14% (n = 42); however, similar percentages of experienced and recent graduates wished they learned more at 34% (n = 48) and 35% (n = 25), respectively; 39% (n = 55) and 35% (n = 25) thought their training to be deficient. Over 40% of practitioners felt that their training was deficient in relation to lingual appliances (n = 96) and aligner therapy (n = 101); 36% (n = 79) and 35% (n = 75) wished they learned more and only 14% (n = 26) and 11% (n = 22) learned the right amount. Similarly, only 41% (n = 89) were satisfied with their training in adult orthodontics. Fifty-eight percent (n = 81) and 59% (n = 42) of established and recent practitioners, respectively, felt their training was deficient or wished they had learned more in this respect. The management of obstructive sleep apnoea received the lowest levels of respondent satisfaction with only 27% (n = 58). Seventy-two percent (n = 156) and 74% (n = 160) learned the right amount in relation to the management of hypodontia and facial deformity/orthognathic, respectively, while 23% (n = 50) and 20% (n = 43) wished they learned more.

## Orthodontics within the NHS

There were generally low levels of satisfaction with training in relation to working with orthodontic therapists (24%, n = 52), understanding of NHS contracts (11%, n = 23) and commissioning of NHS services (11%, n = 24) with no respondents feeling that they had learned more than then they needed to in any of these categories (Table 3).

## Knowledge and skill deficits

Based on free-text responses (Questions 9–11), knowledge deficiency was most frequently reported with aligner systems (28%, n = 61) across all respondents. Adult orthodontics, lingual appliances, commissioning and NHS orthodontics, and temporary anchorage devices were also viewed as more problematic with slight variation between recent and established practitioners (Table 3). The most common self-reported reported skill deficiency related to temporary anchorage devices (24%, n = 52). Other areas of concern included lingual orthodontics and aligner therapy, as well as wire bending (Tables 4–6).

**Table 4.** Subjects that respondents wished they had learned more about.

Overall	Recent Graduates	Established Practitioners
Aligner systems (28%, n=61)	Aligner appliances (37%, n=26)	Aligner systems (25%, n=35)
Adult orthodontics (25%, n=54)	Lingual appliances (35%, n=25)	Adult orthodontics (24%, n=34)
Lingual appliances (24%, n=52)	Commissioning/NHS orthodontics (27%, n=19)	Lingual appliances (19%, n=27)
Commissioning/NHS orthodontics (21%, n=46)	Adult orthodontics (25%, n=18)	Commissioning/NHS orthodontics (19%, n=27)
Temporary anchorage devices (18%, n=40)	Temporary anchorage devices (20%, n=14)	Temporary anchorage devices (18%, n=26)

**Table 5.** Skills that respondents wish they had gained to a greater extent.

Overall	Recent graduates	Established practitioners
Temporary anchorage devices (24%, n=52)	Temporary anchorage devices (32%, n=23)	Lingual appliances (22%, n=31)
Lingual appliances (23%, n=50)	Aligner appliances (27%, n=19)	Temporary anchorage devices (20%, n=28)
Aligner appliances (21%, n=46)	Lingual appliances (25%, n=18)	Aligner systems (19%, n=27)
Wire bending (12%, n=27)	Wire bending (14%, n=10)	Wire bending (11%, n=15)
Running a business (8%, n=18)	Ideal or compromised treatment planning (11%, n=8)	Adult orthodontics (9%, n=13)

**Table 6.** Subject areas in which respondents considered training was excessive.

Overall	Recent graduates	Established practitioners
Research project/MSc (10%, n=21)	Cellular biology (10%, n=7)	Research project/MSc (9%, n=13)
Cellular biology (6%, n=13)	Research project/MSc (10%, n=7)	Cephalometry (5%, n=7)
Embryology (4%, n=9)	Embryology (8%, n=6)	Cellular biology (4%, n=6)
Headgear/extra-oral traction (4%, n=9)	Headgear/extra-oral traction (6%, n=4)	Tip-edge (3.5%, n=5)
Tip-edge (4%, n=9)	Tip-edge (6%, n=4)	Headgear/extra-oral traction (3.5%, n=5)

**Figure 1.**
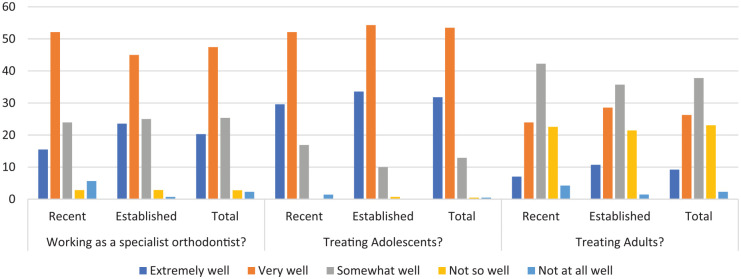
How well did training prepare you for. . . working as a specialist orthodontist, treating adolescents and treating adults?

In terms of how training prepared respondents for working as a specialist orthodontist the overall satisfaction with training was high with 68% (n=147) feeling either ‘extremely well’ or ‘very well’ prepared (Figure 1). Satisfaction rates were, however, markedly lower in relation to training in adult versus adolescent orthodontics (Figure 1).

**Figure 2.**
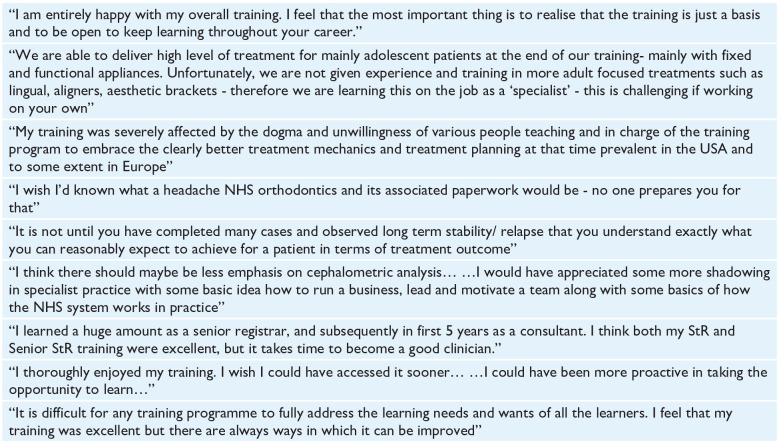
Sample of representative additional comments.

Eighty-six respondents (40%) took the opportunity to leave free-text comments (Figure 2). Fifty described their training in a positive light. Of these, 25 went on to acknowledge limitations relating to the timing of training or environment and 12 suggested areas for improvement. Fourteen made suggestions to improve training with the most frequent suggestions for improvement being inclusion of lingual and aligner appliances (n = 8), additional training in management (n = 5) and development of a period of training to prepare for primary care orthodontics such as a mentoring scheme or vocational training type post (n = 4). Six respondents alluded to their training in a negative way and 13 made comments not relating to their training but mainly about life post-qualification. Negative comments chiefly concerned local ‘politics’ within departments (n = 4).

## Discussion

Generally, respondents were very satisfied with their orthodontic training; particular areas of strength appear to reside in teaching of theoretical concepts, allied to teaching of fixed appliances, removable appliances and removable retainers. Based on allied free-text comments, there appears to be a recognition that postgraduate training stimulates a lifelong commitment to learning and incremental developmental founded on sound clinical principles. There were some reservations concerning teaching of specific aspects including TAD placement, fixed retention, lingual orthodontics, inter-proximal reduction and aligner therapy. These areas place an onus on practical, hands-on teaching allied to espousal of proprietary techniques, which can be less accessible but also often requires adoption of new technologies (Seehra et al., 2017).

Concerns in relation to practical teaching of fixed retention is noteworthy. This finding may again reflect systemic issues whereby limited use of fixed retention is ingrained within selected academic departments. This approach, however, is incompatible with emerging evidence alluding to superiority of fixed retention relative to removable retainers in the medium- to longer- term (Al-Moghrabi et al., 2018; Schütz-Fransson et al., 2019). This discrepancy relates to a decline in compliance over time stemming from lack of supervision following discharge, independent decision-making and limited understanding of the rationale for retention (Al-Moghrabi et al., 2018). It is also interesting to speculate whether this dissatisfaction with teaching concerning retention will ultimately affect practitioner behaviour and protocols, as certification courses concerning retention are certainly less accessible than teaching related to bespoke appliances and technologies. Interestingly, a recent survey of BOS members which highlighted a reduced predilection to extract premolars as part of orthodontic treatment, indicated that this trend was not mirrored by an increased provision of fixed retention (Fleming et al., 2018).

Since many of the respondents completed training there has clearly been progression within the speciality with evolving knowledge and evidence bases, and development of novel appliances and techniques. Some of the specific topics considered in the survey were unlikely to have been in vogue when some of the established practitioners surveyed completed their training and have been introduced and indeed become mainstream during their practicing career. The most notable potential examples of this include temporary anchorage devices, aligner and lingual systems. Incidentally, these approaches represent the areas that all respondents had reservations in relation to. Moreover, the use of treatment modalities (including headgear and TipEdge™) has declined in recent years (Keim et al., 2014a, 2014b), while the emphasis on other key techniques including wire bending may have reduced somewhat with the advent of the StraightWire system. As such, there is a risk that teaching of certain approaches may become obsolete over time; it is important that considered decisions are made in order to supplant these with progressive and evidence-based approaches to ensure that postgraduate teaching remains current and pertinent.

While orthodontic training is standardised within the SAC curriculum, there is variation in relation to treatment philosophies, appliances and techniques that postgraduate trainees are exposed to. There is, for example, a disparity in relation to exposure to removable aligner therapy with some units offering no exposure or training and others providing postgraduates with personal cases to treat throughout their training. Limitations and delays in the introduction of such appliances and techniques may relate to financial constraints and systemic issues. Crucially, techniques such as lingual orthodontics and aligner therapy are not routinely offered with the NHS. As such, there may be systemic constraints and ethical considerations in providing this treatment to a select group of patients. Clearly, there can also be reticence among academics and practitioners to adopt new technology, often correctly reflecting a lack of underpinning evidence to support the use of often heavily marketed products (Seehra et al., 2017). Notwithstanding this, there is an onus on academics and clinical teachers to espouse best current practices, ideally predicated on supporting evidence, where possible.

Teaching in relation to adult orthodontics was frequently reported as deficient with 42% bemoaning a lack of teaching in this respect during postgraduate training both historically and more recently. Current NHS training posts are focused on the management of adolescents within the NHS funding system reflecting recent Commissioning Guidelines (NHS England Chief Dental Officer Team, 2015). Notwithstanding this, adult orthodontics is part of the SAC curriculum despite limited clinical exposure which may often be confined to management of orthognathic or other multi-disciplinary team-based treatments. While this situation does reflect NHS practice, it is at odds with the ever-increasing demand for orthodontics and aesthetic appliances among adults (Nattrass and Sandy, 1995; BOS Admin, 2019).

Respondents reported dissatisfaction concerning the delivery of knowledge on the business aspect of orthodontics including the skillset to lead and manage a team, as well as requisite understanding of the commissioning and contracting of NHS orthodontic services. Currently, the latter years of five-year training pathways are directed at clinical management of more complex multidisciplinary care, but also delivery of management skills required to run a hospital orthodontic department within the secondary NHS care setting. There is no such equivalent for those intent on providing orthodontic care in the primary care setting. Notwithstanding this, complementary courses and mentoring skills addressing these areas are available.

Criticism in relation to excessive training in certain areas was noted with 10% feeling that the research component of their training was excessive while 6% felt teaching in relation to cellular biology was also excessive. The current SAC curriculum refers to ‘undertaking and maintaining a modern evidence-based approach to orthodontic practise’ and having ‘personal research training and experience’ (The Joint Committee for Postgraduate Training in Dentistry and The Specialty Advisory Committee in Orthodontics, 2010). It is therefore expected that trainees either complete at least a Masters level qualification (e.g. MSc, MClinDent, DDS) or publish two articles in peer-reviewed journals relating to work undertaken during the period of training. Although most specialists will not go on to be university academics, there is the expectation that evidence can be critically appraised in order to ensure good evidence-based dentistry is provided to the population. This approach is also reflected in the GDC Principles and Standards (General Dental Council, 2013). Clearly, academic learning and skills are integral to providing evidence-based care ideally underpinning orthodontic decision-making (Madhavji et al., 2011).

While the overall findings from this survey are certainly positive and suggest that orthodontic trainees are generally satisfied, there are undeniably findings which academic directors and educators should digest. Ultimately, decisions will be required concerning any future changes to orthodontic curricula but more specifically to the delivery of teaching on a day-to-day basis. It has been argued that ‘those involved in the education of new dental professionals not to be swayed by the desires of their student consumers, but to keep focused on the wider social picture’ (Lew, 2016). As such, educators must continue to grapple with the social responsibility for training a profession within a public health system to meet the public’s needs, not necessarily the needs of the students.

In terms of limitations, the overall response rate of 20.1% is low. However, this approximates other online surveys assessing the views of dental training (Oliver et al., 2016) and indeed orthodontic treatment planning decisions (Fleming et al., 2018). The survey was distributed through the BOS risking selection bias; however, the society provides access to over 1000 specialist orthodontists, a significant proportion of the estimated 1400 GDC-registered orthodontic specialists (General Dental Council, 2017). Moreover, the sample is representative of the UK orthodontic workforce. A further limitation is the historic nature of some of the data with more experienced practitioners included; clearly, data from the more recently qualified practitioners is of greater current importance. Notwithstanding this, it is important that satisfaction rates among recent graduates is placed in the context of historical data. Furthermore, we were able to report data specific to each group with findings relatively consistent among both subsets.

## Conclusion

The overall satisfaction of BOS members concerning postgraduate orthodontic training is generally high although both recently qualified and established practitioners reflected on a need for enhanced training in specific areas including fixed retention, adult orthodontics, inter-proximal reduction, aligner therapy, lingual appliances and a greater understanding of NHS contracts and commissioning.
